# A PDE Model of Breast Tumor Progression in MMTV-PyMT Mice

**DOI:** 10.3390/jpm12050807

**Published:** 2022-05-17

**Authors:** Navid Mohammad Mirzaei, Zuzana Tatarova, Wenrui Hao, Navid Changizi, Alireza Asadpoure, Ioannis K. Zervantonakis, Yu Hu, Young Hwan Chang, Leili Shahriyari

**Affiliations:** 1 Department of Mathematics and Statistics, University of Massachusetts Amherst, Amherst, MA 01003, USA; nmirzaei@umass.edu (N.M.M.); yuhu@umass.edu (Y.H.); 2 Department of Radiology, Brigham & Women’s Hospital, Harvard Medical School, Boston, MA 02115, USA; ztatarova@bwh.harvard.edu; 3 Department of Mathematics, The Pennsylvania State University, University Park, PA 16802, USA; wxh64@psu.edu; 4 Department of Civil and Environmental Engineering, University of Massachusetts, Dartmouth, MA 02747, USA; nchangizi@umassd.edu (N.C.); aasadpoure@umassd.edu (A.A.); 5 Department of Bioengineering, UPMC Hillman Cancer Center, University of Pittsburgh, Pittsburgh, PA 15219, USA; ioz1@pitt.edu; 6 Department of Biomedical Engineering, Oregon Health & Science University, Portland, OR 97239, USA; chanyo@ohsu.edu

**Keywords:** breast cancer, MMTV-PyMT mouse model, tumor microenvironment, partial differential equation, sensitivity analysis, immune cell influx, finite element method

## Abstract

The evolution of breast tumors greatly depends on the interaction network among different cell types, including immune cells and cancer cells in the tumor. This study takes advantage of newly collected rich spatio-temporal mouse data to develop a data-driven mathematical model of breast tumors that considers cells’ location and key interactions in the tumor. The results show that cancer cells have a minor presence in the area with the most overall immune cells, and the number of activated immune cells in the tumor is depleted over time when there is no influx of immune cells. Interestingly, in the case of the influx of immune cells, the highest concentrations of both T cells and cancer cells are in the boundary of the tumor, as we use the Robin boundary condition to model the influx of immune cells. In other words, the influx of immune cells causes a dominant outward advection for cancer cells. We also investigate the effect of cells’ diffusion and immune cells’ influx rates in the dynamics of cells in the tumor micro-environment. Sensitivity analyses indicate that cancer cells and adipocytes’ diffusion rates are the most sensitive parameters, followed by influx and diffusion rates of cytotoxic T cells, implying that targeting them is a possible treatment strategy for breast cancer.

## 1. Introduction

Breast cancer is the most common type of cancer in women and a major public health problem [1]. It accounts for 25% of the new female cancer cases around the world [2], and it was the cause of 43,600 deaths in the United States alone in 2021 [3]. Breast cancer has three major subtypes: human epidermal growth factor 2 positive (HER2+) (70% of patients), HER2- (15–20%), and triple-negative (tumors lacking all three standard molecular markers; 15%) [4]. Based on the stage of the disease, different treatment options are practiced, such as chemotherapy, radiation therapy, surgical removal, or a combination of these [4,5]. However, these treatments can be straining and do not always work. A comprehensive understanding of the biology of cancer as a complex system of interactions is essential for obtaining effective treatments.

The tumor micro-environment has been the focus of many studies, including therapeutic targeting [6,7] and spatial heterogeneity [8,9,10], as many scientists believe that the key to curing cancer lies within tumor micro-environment interactions [11,12]. However, to find effective therapies, many factors should be considered, and in-vivo examinations of the tumor micro-environment and its response to treatments can be expensive and straining for both patients and investigators. Therefore, mice models of breast cancer are prevalent for testing different hypotheses and treatment combinations.

To establish mouse models, many different approaches have been used, including transgenic [13,14,15,16], gene targeting [17,18,19], and RNA interference [20,21,22]. Transgenic mouse models (in which oncogenes can be expressed while other factors, such as tumor-suppressor genes, are muted), are used to study breast cancer and improve our understanding of cancer initiation and progression [23,24,25,26]. For example, using these mouse models, it has been shown that macrophages regulate the cancer progression and formation of a high-density vessel network [27], and the high mobility group box-1 (HMGB-1) proteins play roles in promoting angiogenesis and tumor migration [28]. One of the most commonly used mouse models in cancer research is the mammary-specific polyomavirus middle T antigen overexpression mouse model (MMTV-PyMT) [29,30,31]. Specifically for breast cancer, this mouse model has been utilized successfully and qualifies as a transgenic approach [32,33].

At the same time, mathematical modeling of cancer development and tumor micro-environment offers insights and can be used in discovering new treatments [34,35,36,37,38,39,40,41,42,43,44,45,46,47]. As the complex spatial cell-to-cell interactions in the tumor micro-environment has attracted many experimental studies, a more thorough mathematical model can help scientists gain a better insight into the mechanisms of cancer growth. Many mathematical models using partial differential equations (PDE), which allow the integration of spatial information into the governing differential equations, have been developed to study health problems, such as atherosclerosis, Alzheimer’s disease, and COVID-19 spread [48,49,50,51,52]. Since the pioneering work of Iwata et al. [53] that introduced a PDE dynamical model for the metastatic evolution of an untreated tumor, researchers have developed several PDE models for cancer [54,55,56,57]. For example, for breast cancer, a PDE model with a 1D domain was proposed in which a predetermined population of tumor cells interacts with a fixed population of macrophages in a tissue-culture experiment [58]. In another study, a 2D spatial hybrid model was suggested, where an ordinary differential equation (ODE) described the dynamics of each immune cell, and a PDE described the evolution of the average substances released by the tumor cells [59]. Lai et al. also developed a system with eight variables, including cell populations of macrophages and T cells, to model the combination therapy for breast cancer [60].

Biological tissues have specific mechanical properties which can affect the growth and deformation caused by cell proliferation and movement in addition to the important cellular and molecular interactions. Since the pioneering works of Y.C. Fung on modern biomechanics [61,62,63,64], many specific tissue constitutive models have been proposed [65,66,67,68]. As mechanical properties of tumors can differ depending on the hosting tissue, different mechanical models have been developed for different cancer types. In particular, breast tumors have been treated as porous media and fluid-like tissue. For example, Frieboes et al. modeled breast tumor tissues as porous media and implemented a model that incorporates the interplay between the local drug, oxygen, and nutrient concentrations [69]. On the other hand, Friedman and Hu argued that due to a high content of the extracellular fluid in mammary glands, breast tumors can be modeled by the Stokes equation [70], and their method has been successfully applied to many studies [71,72,73,74].

This paper proposes a system of PDEs consisting of 15 variables coupled with the Stokes equation governing the tumor micro-environment. The coupling is performed through the velocity involved in the convection terms of the PDE system. This study improves our previous model developed via a system of ODEs [75]. We incorporate the parameter estimations for the ODE system in this paper and extract the PDE-specific parameters from the literature. Spatially dependent initial conditions for each cell type are obtained from an MMTV-PyMT mouse model by using multiplexed immunohistochemistry and investigating certain combinations of biomarkers such as Epcam, CC3, CD45, CD3, CD4, CD8, CD11c, F4/80, CSF1R, and MHC-II in the experimental domain. The simulations are done via the finite element method (FEM). We first investigate a naive case similar to our experimental data for which we do not consider any immune cell influx. Later, we include an influx of specific immune cells. We study tumor growth, cell and molecules dynamics, the spatial significance of immune cell distributions, and the effect of immune cell influx on cancer and domain growth. Finally, we perform an adjoint-based sensitivity analysis to find the sensitivity of total cancer cells to all the parameters of our model. We comment on the similarity of our results with the ODE study and investigate the biological importance of the PDE-specific parameter sensitivities.

## 2. Materials and Methods

**Figure 1:**
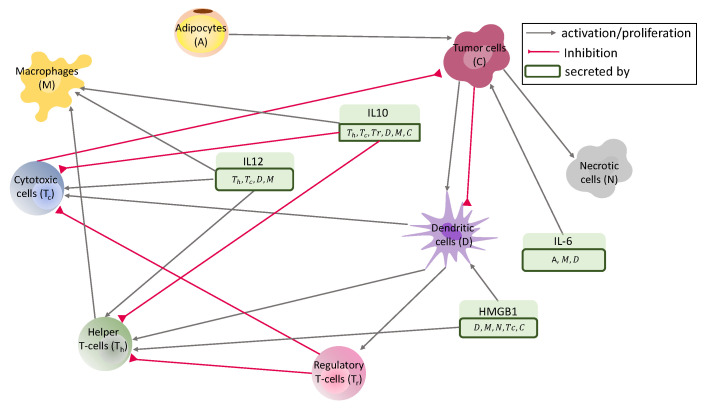
*Interaction network.* Interaction between key cells and molecules.

The interaction network consists of helper T cells, cytotoxic T cells, regulatory T cells, dendritic cells, macrophages, adipocytes, cancer cells, and necrotic cells and molecules, including HMGB1, interleukin-6, interleukin-10, and interleukin-12, see Figure 1.

The effect of nutrients and metabolites on tumor growth has been studied extensively. They can effect cancer cell growth directly or through the immune cells such as T cells [76,77,78]. However, to avoid too much complexity, we neglect the effect of nutrients and metabolites in this study.

### 2.1. PDE System

While ODE models give valuable information about cell and molecule dynamics, they cannot capture the spatial effect of cell-cell and cell-molecule interactions. For a more realistic model of the cancer evolution, we considered a Reaction-Diffusion-Advection (RDA) PDE system, in which the reaction terms come from the ODE system mentioned above [75]. The advection was directed by a velocity field acquired from the mechanical properties of the tumorous tissue and triggered by the cells’ growth in the cancer micro-environment. We assumed that naive T cells and naive macrophages get activated outside the tumor micro-environment [79]. Therefore, the region’s deformation and growth depend on the model’s other cell types: helper, cytotoxic and regulatory T cells, naive and activated dendritic cells, activated macrophages, cancer cells, necrotic cells, and adipocytes.

We used a common approach of modeling cells’ and molecules’ movements and diffusion rates in the tumor [48,80,81,82,83,84]. Given the significant difference between the size of cells and molecules, in these studies, it was assumed that the domain volumetric change is only affected by the movement of cells inside of the tumor microenvironment and not the molecules. We, therefore, only considered advective terms in the PDEs for cells in the tumor and not the molecules or cells outside of the tumor. These assumptions lead to the following system of PDEs:

(1) $$\begin{array}{c} \frac{d[X_{i}]}{dt}+b_{i}\nabla \cdot \left(v\left[X_{i}\right]\right)=D_{i}\Delta \left[X_{i}\right]+f_{i}, \end{array}$$

where $\left[X_{i}\right]$ for i=1,⋯,15 corresponds to the 15 variables form the ODE system given in [75]. Table 1 indicates the list of the variables of the ODE model and their corresponding names in the PDE model. The operators ∇· and Δ are the divergence and Laplacian, respectively. The vector v represents the advection velocity, and $D_{i}$ is the diffusion coefficient for the cell or molecule $\left[X_{i}\right]$. The function $f_{i}$ represents the right-hand side (biochemical reactions) of the *i*-th ODE equation, and 

(2) $$\begin{array}{c} b_{i}=\left\{\begin{array}{c} 0\;\mathrm{if}\;i=\{1,7\}\cup \{12,\cdots ,15\},\\ 1\;\;\mathrm{otherwise}. \end{array}\right. \end{array}$$

To exclude the effect of molecules and outsider cells on volumetric changes, indices i=1,7 correspond to the naive T cells and naive macrophages, and {12,⋯,15} correspond to the molecules. Similarly,

(3) $$\begin{array}{c} D_{i}=\left\{\begin{array}{c} 0\;\mathrm{if}\;i=\{1,7\},\\ d_{i}>0\;\;\mathrm{otherwise}. \end{array}\right. \end{array}$$

Because we did not model the diffusion outside the tumor micro-environment, the equations for naive T cells and naive macrophages remained as ODEs. Furthermore, we assumed that all cells in the model have approximately the same volume and surface area, and hence the same diffusion coefficient [50,81,83].  

(4) $$\begin{array}{ccc} d_{i} & = & 3.6\times 10^{-8}\;\frac{{\mathrm{cm}}^{2}}{h}\;\mathrm{for}\;i=\left\{2,3,4,5,6,8,9,10,11\right\}, \end{array}$$

(5) $$\begin{array}{ccc} d_{i} & = & 3.3\times 10^{-3}\;\frac{{\mathrm{cm}}^{2}}{h}\;\mathrm{for}\;i=\left\{12\right\}, \end{array}$$

(6) $$\begin{array}{ccc} d_{i} & = & 5.2\times 10^{-5}\;\frac{{\mathrm{cm}}^{2}}{h}\;\mathrm{for}\;i=\left\{13,14,15\right\}. \end{array}$$

These small constant diffusion rates for cells help with smoothness while avoiding too much complexity in the sensitivity analysis. This is a common simplification [48,80,81,82,83] that leads to advection-dominated PDEs for cells.

#### Boundary Conditions

Immune cell infiltration in cancer studies has important prognostic implications [85,86,87,88,89]. In order to control and analyze the rate and intensity of infiltration of these cells, we used Robin boundary conditions through the tumor’s outer boundary, which has been used to model the influx rate of cells in tumors [90,91]. Namely,

(7) $$\begin{array}{c} \frac{\partial [X_{i}]}{\partial n}+\alpha_{i}\left(\left[X_{i}\right]-\left[{X_{i}}^{*}\right]\right)=0,\;\mathrm{on}\;\partial \Omega \left(t\right) \end{array}$$

where ∂Ω(t) is the boundary of the tumor at time *t*, the vector n is the outward unit normal vector to the boundary, and $\alpha_{i}$ is the influx rate and is only nonzero for immune cells; we considered zero flux boundary conditions for the rest of the cells and molecules. The quantity $\left[{X_{i}}^{*}\right]$ pertains to the maximum levels of immune cells in lymph nodes and blood.

### 2.2. Mechanical Model

Many mathematical models of tumors assume that the tissue is a porous medium [92,93,94]. Then, due to the high permeability of macrophages and other cells [95,96], one can treat them as a low-speed flow through the porous tissue with the advection velocity v [90]. On the other hand, some studies consider the tumor as fluid without a solid structure interaction [72,80,97]. Especially in the case of breast cancer, they argue this approach is reasonable since the tumor is mainly confined in the mammary gland, which has a high content of the extracellular fluid. For this study, we considered the breast tumor as a fluid, and we followed the method introduced in [70]. We also assumed that the changes in the volume and surface area of the cells in the cancer micro-environment were negligible and the domain was an incompressible, continuous fluid, with no voids inside. Hence, the sum of the densities of all cells remained constant [48,80]. If we take I={2,3,4,5,6,8,9,10,11} (with indices 1 and 7 missing on purpose) to be the set of indices corresponding to the cells present in the tumor micro-environment, then

(8) $$\begin{array}{c} \sum_{i\in I}\left[X_{i}\right]=\mathrm{constant}. \end{array}$$

Since the breast tissue is mainly decomposed into water, lipid, and protein with corresponding mass densities of 1, 0.924, and 1.35 g/cm^3^ [98], for simplicity, we assume that the constant in (8) is on average 1. Hence, summing both sides of (1) over i∈I and applying (8) implies:

(9) $$\begin{array}{c} \nabla \cdot v=\frac{\sum_{i\in I}f_{i}}{\sum_{i\in I}\left[X_{i}\right]}. \end{array}$$

For modeling the fluid-like behavior of the breast tumor, we use the Stokes equation:

(10) $$\begin{array}{c} \nabla Q=0\;\mathrm{in}\;\Omega (t),\;t>0, \end{array}$$

where Ω(t) is the tumor region at the time *t* and

(11) $$\begin{array}{c} Q=\nu \left(\nabla v+{\left(\nabla v\right)}^{T}\right)-\left(p+\frac{2}{3}\nu \mathrm{div}\left(v\right)\right)I, \end{array}$$

with ν and *p* being viscosity and the hydrostatic pressure. We assume that cell-cell adhesion force on the boundary of a tumor keeps the domain connected [99,100]. Taking γ to be this force, κ to be the point-wise mean curvature of the boundary, and n to be the unit outward normal vector for the boundary Γ(t), we have the following boundary condition:

(12) $$\begin{array}{c} Qn=-\gamma \kappa n\;\mathrm{on}\;\Gamma (t),\;t>0. \end{array}$$

Additionally, we assumed the kinematic boundary condition:

(13) $$\begin{array}{c} v\cdot n=V_{n}\;\mathrm{on}\;\Gamma \left(t\right),\;t>0, \end{array}$$

where $V_{n}$ is the velocity of the free boundary Γ(t) in the direction of n. Finally, since the problem has a 6 dimensional kernel in 3D (and a 3 dimensional one in 2D) of the form $v_{0}=a+b\times x$, with a and b being arbitrary vectors and x being the deformation vector, we consider the constraints

(14) $$\begin{array}{c} \int_{\Omega (t)}v\;dx=0,\;\int_{\Omega (t)}v\times x\;dx=0, \end{array}$$

to exclude rigid body movements such as translation and rotation. The system consisting of Equations (9), (10) and (12)–(14) has a unique solution [101].

There are no precise reports of the values ν and γ in the literature. Rianna and Radmacher [102], for thyroid cancer ν≈ 350,000$\;\frac{\mathrm{mg}}{\left(\mathrm{mm})\cdot (s\right)}$, and Sancho et al. report an approximate value of $200\;\frac{\left(\mathrm{mg})\cdot (\mathrm{mm}\right)}{s^{2}}$ for general cell-cell adhesion force (γ) in-vitro [103]. For this study, we scaled the problem so we can take ν=1, and this scaling would result in $\hat{\gamma }∼O\left(10^{-4}\right)$. Since we worked with mouse data, we tended to go with the lower bound. We, therefore, took the scaled $\hat{\gamma }$ to be exactly $10^{-4}$. However, we acknowledge this ad-hoc estimation as a limitation of our study.

### 2.3. Data of the Model

#### 2.3.1. Mouse Model and Experiments

Tumors from naïve mouse mammary tumor virus-polyoma middle tumor-antigen (MMTV-PyMT) mice were harvested at 3–5 mm in diameter (early), and 12–15 mm in diameter (late) size and were prepared as formalin-fixed, paraffin-embedded (FFPE) samples. The 4–5 μm tumor sections were stained using multiplex immunohistochemistry (mIHC)—a process of serial immunostaining, imaging, and stripping—to assess a range of markers with specific staining patterns being cross-validated by using cyclic immunofluorescence (cycIF). Each mIHC image was analyzed by segmenting individual cells and calculating marker positivity for each segmented cell. For this study, we developed a comprehensive mouse-specific readout panel including proteins such as Epcam, CC3, CD45, CD3, CD4, CD8, CD11c, F4/80, CSF1R, and MHC-II to interrogate a broad range of tumor and tumor microenvironment states and functions. Table 2 lists cell classification based on biomarker combination. Notice that since we consider all subtypes of activated macrophages as one variable, we have considered two combination biomarkers for it. For regulatory T cells, we assume T cells that are neither cytotoxic nor helper are regulatory. Moreover, the combination Epcam(−) CD45(−) may include fibroblasts, endothelial cells and pericytes, and adipocytes. However, here, we assume it only refers to adipocytes.

The tumors were derived from immunocompetent MMTV-PyMT mice with spontaneously growing tumors that mirrored the morphology and aspects of progression of human breast cancer [30]. For the details of the experiments, please see Appendix B. Table 2 explains the biomarker combinations.

#### 2.3.2. Preparation of Initial Conditions

**Figure 2:**
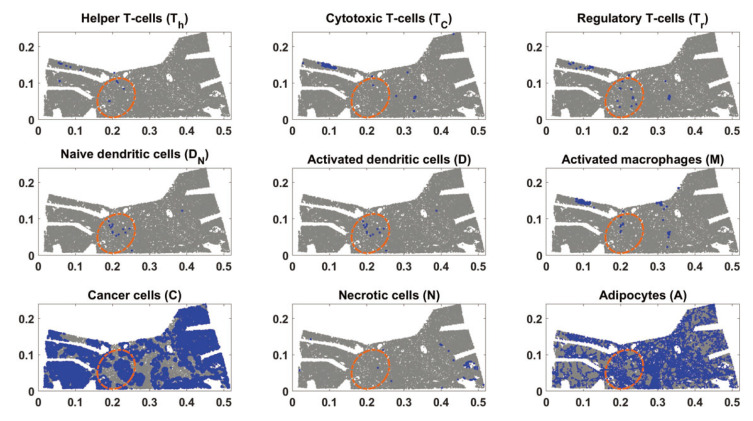
Nine figures showing the position of the chosen elliptical domain compared to each cell type. Blue dots represent a single cell of the corresponding cell type, and gray dots are the rest.

To avoid the instability caused by an irregular boundary, we used an elliptical domain containing all the model’s cell types. Figure 2 shows the mathematical region superimposed on the experimental domain. Single cell-type locations were determined by the bio-marker combinations given in Table 2.

We used a triangular mesh on the mentioned elliptical domain. We assigned a discontinuous Galerkin function space of degree zero to this mesh. Then, for each cell type, we defined piece-wise functions as follows:

(15) $$\begin{array}{c} X_{i}\left(x\right)=\sum_{j=1}^{m}\omega_{i}\left(T_{j}\right)\chi_{j}\left(x\right),\;\mathrm{for}\;i\in I, \end{array}$$

where $\omega_{i}\left(T_{j}\right)$ is the number of the cell type $\left[X_{i}\right]$ in the triangle $T_{j}$. The function $\chi_{j}\left(x\right)$ for a domain point *x* is a characteristic function defined by:

$$\chi_{j}\left(x\right)=\left\{\begin{array}{c} 1\;\mathrm{if}\;x\in T_{j},\\ 0\;\mathrm{Otherwise}. \end{array}\right.$$

**Figure 3:**
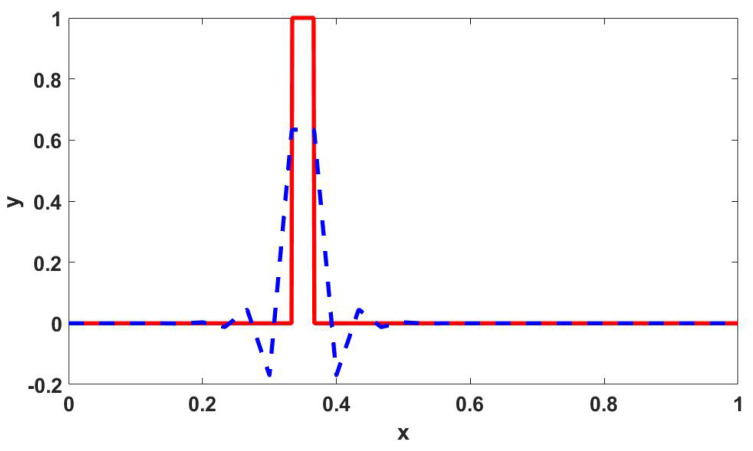
*Solid red:* A discontinuous function. *Dashed blue*: The projection onto a finite element space with linear Lagrangian bases.

We project the function defined in (15) onto a function space with linear Lagrangian elements to get a continuous representation for the initial state of each cell type. However, this projection might result in non-smoothness. Figure 3 shows a one-dimensional case of this issue.

To prevent the propagation of such anomalies in our simulation, we flatten the negative values and then introduce a primary diffusion step to smooth things out. After this step, we non-dimensionalize each field by dividing it by its maximum value across the domain. This concludes the initial condition preparation. See Appendix C and Figure A2 for a visualization of these steps and the final products, which we use as the initial conditions for solving the system (1).

The parameters involved in the reaction term of Equation (1) are evaluated based on an estimation method proposed in a previous study [75]. In that study, we had time-course data for three PyMT mice, and we performed a least-squares optimization to obtain the reaction parameters. Here, we consider constant initial conditions for HMGB1, IL-12, IL-10, and IL-6; these constants are taken to be the initial values of mouse 1 from the ODE model presented in [75]. Finally, as mentioned before, Equations (1)–(3) for naive T cells and naive macrophages are actually ODEs. Therefore, we take their initial conditions to be a constant of 1.

## 3. Results

### 3.1. No Influx

**Figure 4:**
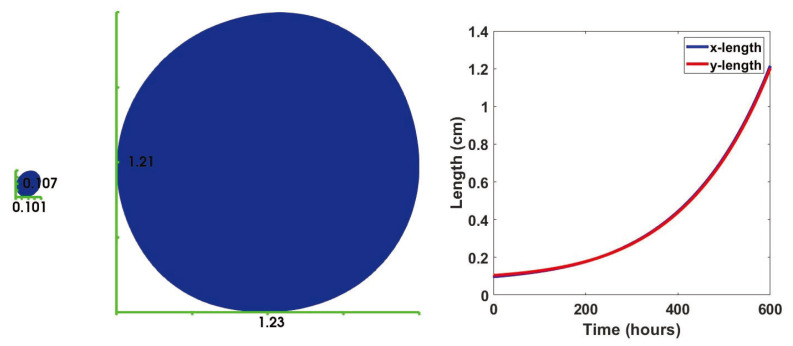
Comparison between the dimensions of the tumor at t=0 h versus t=600 h. The graphs show the time evolution of the bounding box dimensions.

We used a finite element method to simulate our results (see the Appendix D). We started by investigating a case with no influx source for the immune cells. In other words, we tooko $\alpha_{i}=0$ in (7). Using the initial conditions from the fourth column of Figure A2, constant initial conditions for cytokines (taken from [75]) and naive T cells and naive macrophages (taken to be 1), we solved the discrete mechanical and biological problem discussed in Appendix D. Figure 4 shows the change in the length and width of the bounding box containing the domain. For this case, we carried out our simulations for 600 h (25 days). It is worth pointing out that the extractions of early and late tumors mentioned in Section 2.3.1 were about three weeks apart. The almost circular form of the domain is due to the effect of the boundary condition (12), and the deformation generally happens in the direction of the mean curvature. Even though for this problem, we have conveniently picked an elliptic reference region; cancer scientists commonly observe a blob-shaped final results [104,105,106]. The small deviations from a fully circular region in Figure 4 and the rest of this paper is the result of the competition between deformation by the reaction term (9) and the boundary term (12) throughout the region.

**Figure 5:**
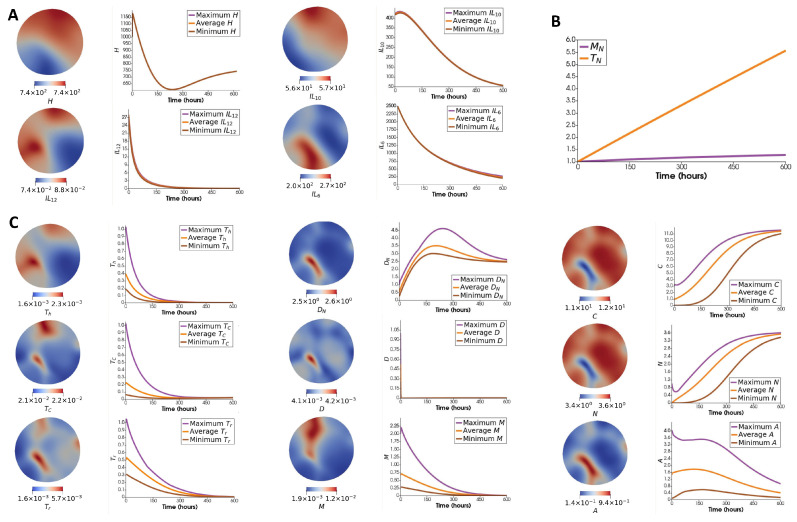
Results with no flux of immune cells. (**A**) *Column 1*: Spatial distribution of cytokines. *Column 2*: Maximum, average, and minimum concentration (ng/mL) of each cytokine over the whole domain with respect to time. (**B**) Evolution of naive T cells and naive macrophages. (**C**) *Column 1*: Spatial distribution of cell types. *Column 2*: Maximum, average, and minimum number of each cell type over the whole domain with respect to time.

Figure 5A describes the spatial and evolutionary behavior of cytokines, and Figure 5B shows the evolution of two naive cell types excluded from the tumor micro-environment. Figure 5C shows the spatial distribution of each cell type in the tumor micro-environment next to their maximum, average, and minimum over the whole domain with respect to time.

**Figure A1:**
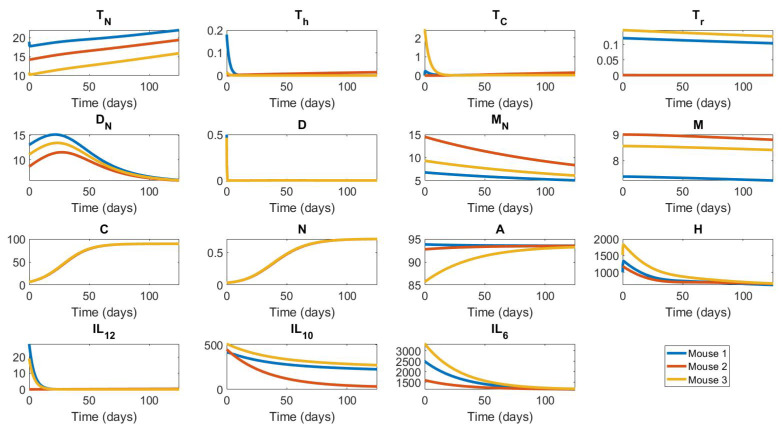
Cells and molecule dynamics from the ODE model [75].

**Figure A2:**
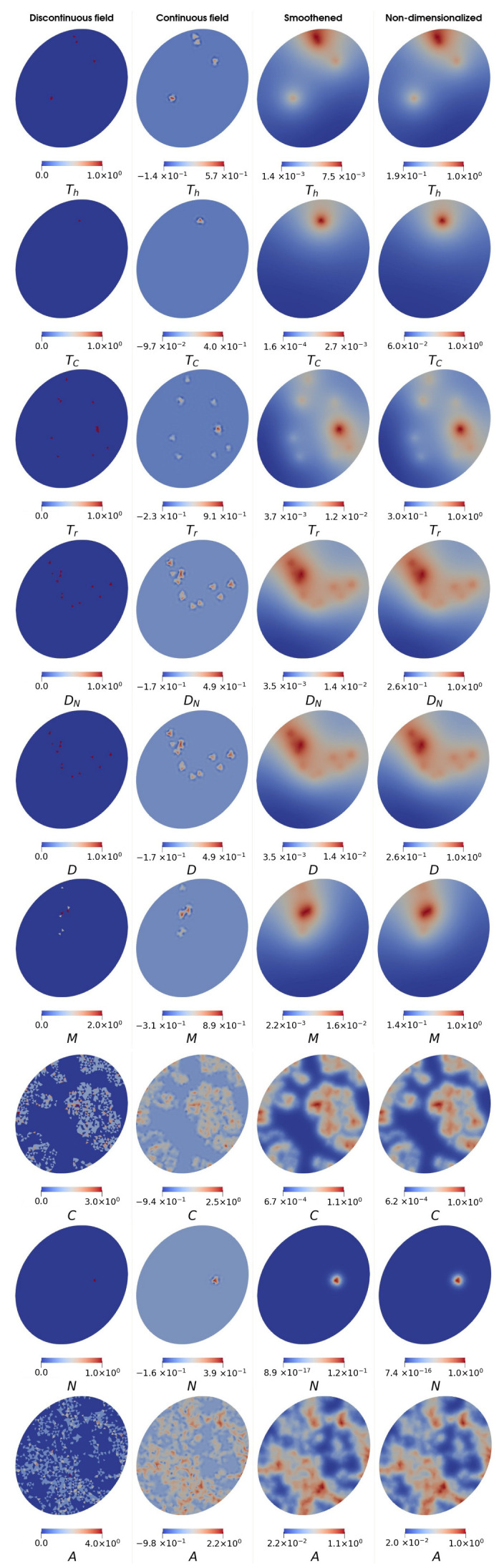
*Column 1*: Discontinuous fields. *Column 2*: Projection onto a function space with linear Lagrangian elements. *Column 3*: Smoothened fields via diffusion. *Column 4*: Non-dimensionalized fields.

This result shows that most of the cell types deplete in time except cancer, necrotic and naive dendritic cells (Figure 5). The qualitative behavior of most of the results is comparable with the ODE case, especially mouse 1; see Figure A1 in Appendix A. The most significant difference is the behavior of naive macrophages. The naive macrophages have a strictly decreasing dynamic in the ODE paper, unlike here. The reason might be connected to the slower depletion of cytokines IL10, IL12, and helper T cells, which are the main contributors to the inhibition of naive macrophages. Additionally, HMGB1 has a sharp increase, followed by a decrease in our ODE results. However, the decrease is not monotonic, and in mouse 2, it stops for a short while. Additionally, the timescale of this simulation is much smaller than the ODE paper. To summarize, naive macrophages and HMGB1 are the only fundamentally different results from the behaviors observed in our ODE paper. For the rest of the variables, the changes in the total populations/densities are similar to their dynamics observed in the ODE model. Biologically, the observed decreasing behavior of the immune cells is attributed to cancer cells’ ability to evade identification and invasion by host immune responses in later stages of cancer [107,108,109].

**Figure 6:**
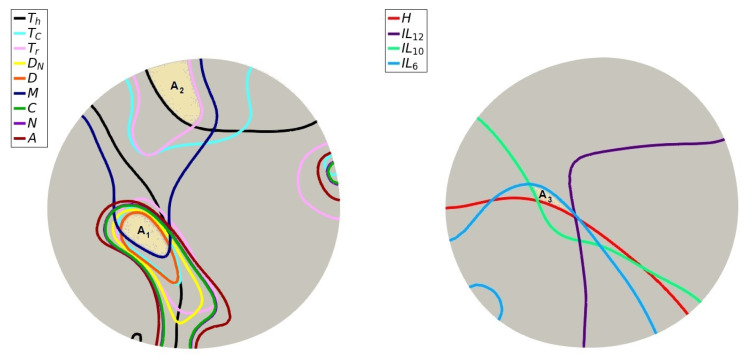
(**Left**): Level-curves indicating the mean value of each cell type at t=600. (**Right**): Level-curves indicating the mean value of each molecule at t=600. Areas $A_{1}$ and $A_{2}$ correspond to the regions with the most and second-most immune cell intersections, respectively. Area $A_{3}$ corresponds to the region with the highest cytokine intersections.

An ODE model fails to capture the significance of the spatial distribution of cells and cytokines. To see how that affects cells and molecules, we studied their level-curve plots. Figure 6 show the contours for cells (left) and molecules (right) separating the regions with values above and under the average of their corresponding field at t=600. Colored areas have the highest number of intersections within the plot. Comparing Figure 5C and Figure 6, we can see that the cancer cells have a minor presence in the area with the most overall immune cells (region $A_{1}$). There is also an intersection between regulatory and cytotoxic T cells with macrophages in the $A_{2}$ region, which might be the reason for the slightly decreased cancer population in the corresponding location. The intersection between molecules (region $A_{3}$) happens too close to the shaded region to leave a discernible spatial footprint.

**Figure 7:**
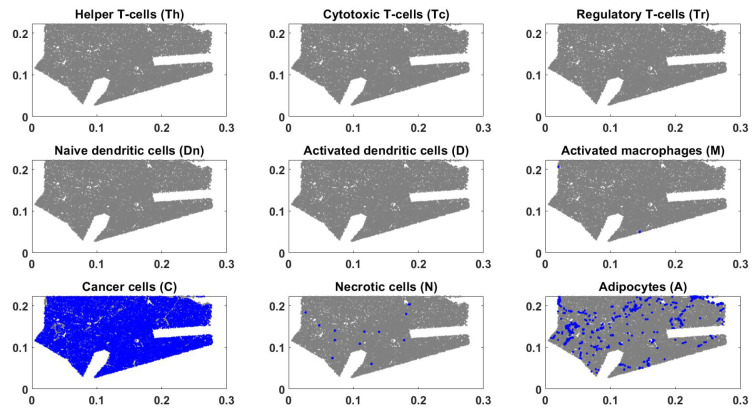
Nine figures showing each cell type in the mouse model. Blue dots represent a single cell of the corresponding cell type, and gray dots represent the rest.

We compared the simulation results with the experiments. The late foldout of the mouse model used in this study is given in Figure 7. The late foldout is extracted under a naive regime as well, i.e., no treatment has been applied to the mouse model (see Section 2.3.1). Therefore, immune recruitment and infiltration are negligible. Even though it is impossible to show exactly where our simulation domain is located due to the lack of a common reference frame, we can see a good agreement in the overall qualitative behavior of the cell populations. We can see a significant increase in the number of cancer cells compared to Figure 2, while most of the other cell types are depleted. Adipocytes are settled at lower levels, just like the mathematical model’s results. The major differences between the mathematical model’s results and the late foldout are the naive dendritic and necrotic cells. Because of the simplicity and lack of data, the mathematical model does not include metabolites and nutrients, which play a crucial role in necrosis. Here, the only source of necrosis is the death of cancer cells, so they inevitably follow the same trend as cancer cells. Additionally, since HMGB1 acts as a major inhibitor of naive dendritic cells, the mouse model may have a higher level of this molecule.

### 3.2. Immune Cell Influx

**Figure 8:**
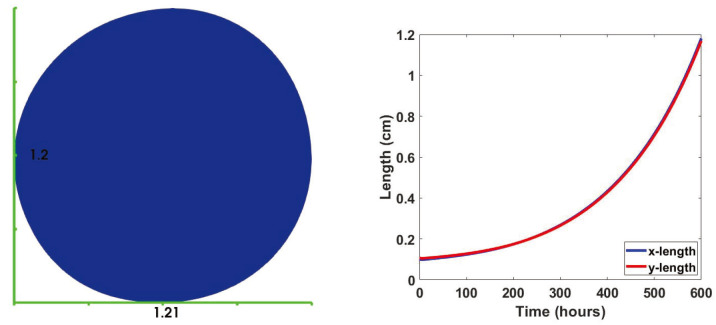
Dimensions of the tumor subject to immune cells influx at t=600 h. The curves show the time evolution of the bounding box dimensions.

In this section, we consider the influx rates $\alpha_{i}=1$ and non-dimensional influx sources $[X_{i}^{*}]=1$ for the helper, cytotoxic and regulatory T cells, naive dendritic cells, and macrophages. We assume no-flux boundary conditions for other cells and molecules. Since activated dendritic cells are differentiated from the naive ones, and this activation happens mostly inside of the tumor micro-environment [110], we assume a no-flux boundary condition for the activated dendritic cells. Figure 8 shows the domain after t=600 h. Compared to Figure 4, the domain is smaller.

**Figure 9:**
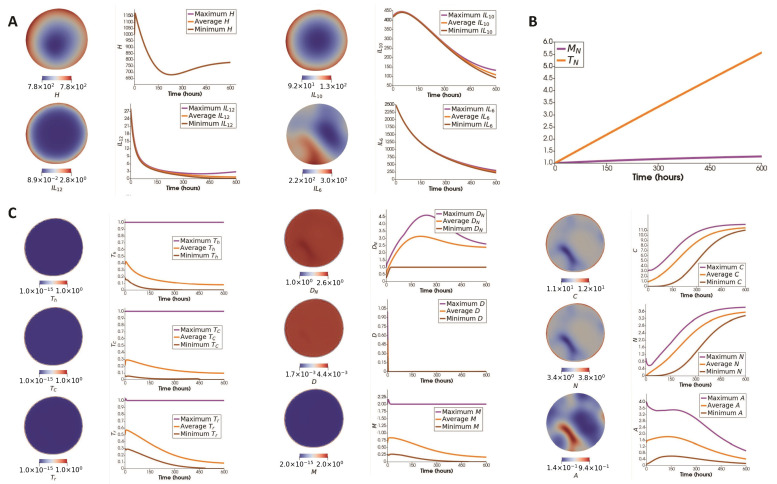
Results with flux of immune cells. (**A**) *Column 1*: Spatial distribution of cytokines. *Column 2*: Maximum, average, and minimum concentration (ng/mL) of each cytokine over the whole domain with respect to time. (**B**) Evolution of naive T cells and naive macrophages. (**C**) *Column 1*: Spatial distribution of cell types. *Column 2*: Maximum, average, and minimum number of each cell type over the whole domain with respect to time.

**Figure 10:**
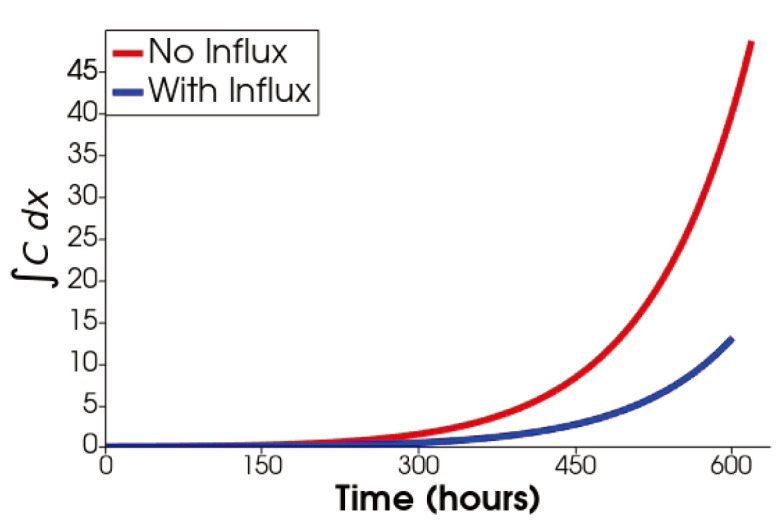
Evolution of the integral of cancer over the domain with and without immune cell influx.

Figure 9 shows the spatial and evolutionary behavior of cytokines and cells. Naive T cells and macrophages do not change much since they are not spatially dependent. However, due to the Robin type boundary condition, we see either a stationary maximum value ($T_{h}$, $T_{C}$, $T_{r}$ and *M*) or a stationary minimum value ($D_{N}$) close to the boundary. The former is because the field values are depleting across the domain and the influx tends to bring it up, and the latter is precisely the opposite. The distribution of dendritic cells follows the same trend as the naive dendritic cells. However, it decreases quickly, just like the no-influx case. Due to the near boundary focus of the cells, the molecules are more intense closer to the boundary. As for cancer cells, it seems that their distribution inside of the region and away from the boundary is similar to what Figure 5 shows but at much lower values. On the other hand, there is a higher intensity close to the boundary. Note that this high-intensity region covers a very tiny area, and in total, there are fewer cancer cells present in the region due to the immune cell influx. This is more evident when we compare the integral of cancer cells over the domain for the two cases (Figure 10).

In the model, $T_{C}$ is considered the primary inhibitor of the cancer cells, and $IL_{6}$ and *A* are the main contributors to their production. Since $T_{C}$ is more intense close to the boundary and $IL_{6}$ and *A* have the same behavior as in the previous case, one might expect to see fewer cancer cells at the boundary. We hypothesize that, even though reactions are decisive in the model, this phenomenon is more because of the cells’ advection direction in the presence of immune cell influx. In other words, cancer cells tend to leave the region. This can be a dangerous trait, given that usually mitotic regions are close to the boundary of the tumor [106,111]. This might explain why some types of breast cancers still metastasize despite therapy and significant immune recruitment.

Similar to the no-influx case, the necrotic cells follow the same pattern as cancer cells. Finally, adipocytes show precisely the same behavior as before. Even though they influence other cells and molecules, their behavior is independent of the other variables. Thus, we observe the same behavior.

### 3.3. Sensitivity Analysis

To investigate the impact of parameters on the cancer population, we performed a sensitivity analysis on all the model parameters for the case with immune cell influx. We applied an adjoint sensitivity analysis for the functional

(16) $$G=\int_{\Omega }C\;dx\;\mathrm{at}\;t=600.$$

**Figure 11:**
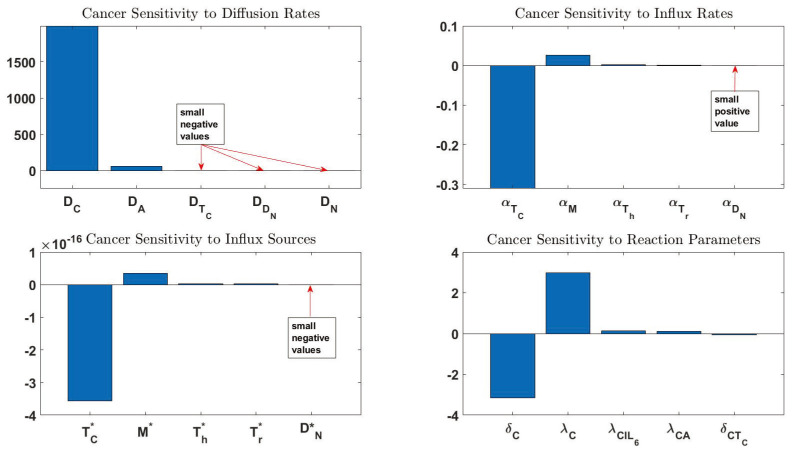
The sensitivity of $\int_{\Omega }C\;dx$ at t=600 to four categories of parameters: diffusion rates, influx rates, influx sources, and the reaction parameters.

We used this method because the model contains a large set of parameters, and the computational cost of adjoint-based sensitivity analysis is almost independent of the number of input parameters. Hence, we can compute sensitivities with respect to numerous parameters or even entire functions. For more information on the mathematical detail of the method, please see [112]. To carry out the sensitivity analysis using adjoint methods, we took advantage of the dolfin-adjoint package [113]. The benefit of this package is that it can be mounted on FEniCS and can record each step of the simulation. Once the adjoint-based sensitivity starts, it automatically steps backward and calculates the sensitivities. Figure 11 shows the top five sensitivity values of (16) to four category of parameters: diffusion rates, influx rates, influx sources, and the rest of the reaction parameters. For a full report of the sensitivity analysis, parameter notations, and definitions, see Appendix E and Table A1.

Interestingly, the top four most sensitive reaction parameters were captured in the same order in the ODE sensitivity analysis [75]. However, despite acknowledging $\delta_{CT_{C}}$ as a sensitive parameter, it was not this high up in the ODE study. This is interesting because $T_{C}$-related PDE parameters also show significant sensitivities, which are negative. This is due to the model’s higher levels of $T_{C}$ after imposing an influx for this cell type.

Moreover, the diffusion rate of cancer cells is one of the most sensitive parameters. In other words, the more motile the cancer cells get, the larger their population becomes. This is because cancer cells will interact more with cells and molecules that promote their proliferation. The diffusion rate of adipocytes is the following most sensitive parameter in this model. Adipocytes directly activate cancer cells, and increasing their motility means more cancer/adipocyte handshakes.

The macrophage-related parameter, $\alpha_{M}$, has a relatively high positive sensitivity value. This means that a greater influx of macrophages leads to more cancer cells. This is due to the fact that macrophages produce $IL_{6}$ and $IL_{10}$, which, respectively, cause cancer cell proliferation and inhibition of cytotoxic T cells. Traditionally, macrophages are the immune system soldiers in charge of clearing target cells. However, many studies have investigated the tumor-promoting capabilities of macrophages [114,115,116,117].

**Figure 12:**
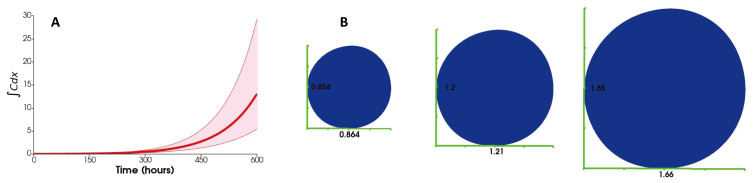
(**A**) The variation of the total number of cancer cells as a result of 10% perturbation of the most sensitive parameters. (**B**) The leftmost circle corresponds to the lower bound, the middle circle corresponds to the thick solid red curve and the right circle corresponds to the upper bound of the graph in (**A**).

Figure 12A shows the result of 10% perturbation of the top 20 most sensitive parameters given in Table A1. We can see a significantly lower number of cancer cells at the lower 5%. Moreover, Figure 12B shows a drastic change in the size of the tumor when the most sensitive parameters are varied.

Since the diffusion of cancer cells and adipocytes are the most sensitive parameters, the results indicate targeting cancer cells motility might be a treatment strategy, as suggested in some other studies [118,119]. Parameters number 3 and 4 from Table A1 are not informative, because they engulf production and death caused by reasons other than the ones we have included in the model. Parameters number 5, 6, and 9 emphasize the importance of cytotoxic T cells in cancer inhibition. Targeting CD8+ for immunotherapy has been discussed extensively in the literature [120,121,122]. As for parameters 7 and 8, we refer to the ODE paper for a detailed discussion about the importance of adipocytes and IL6 in cancer therapy [75].

## 4. Discussion

In this study, we investigated the spatial interaction network of key cells and molecules in the breast cancer tumors of a PyMT mouse model by developing a bio-mechanical system of PDEs. We adopted the critical reactions among cells and molecules from an ODE model of mice breast tumors [75], and the initial conditions were extracted from an MMTV-PyMT mouse model.

Since there was no treatment applied in the experimental mouse model, the recruitment of the immune cells was negligible. We, therefore, first investigated a case with no immune cell influx. We noticed that the domain grows and deforms into a larger blob-shaped region, a shape which is commonly observed in experiments [104,105,106].

The spatial distribution of the model state variables, which are in good agreement with the late foldout experimental results, shows that the regions with the higher number of immune cells have much fewer cancer and necrotic cells. The only differences between the late foldout cells distributions in the mathematical model and experimental results are the number of necrotic cells and naive dendritic cells. We hypothesize that the former is due to the lack of nutrients and metabolites in the mathematical model, which affects necrosis [123,124]. The latter is possibly due to the level of HMGB1 (a major activator of naive DCs) in the mouse model compared to the mathematical model.

Moreover, we modeled an influx for immune cells through Robin boundary conditions. The results indicate a lower growth rate of the domain with influx compared to the no-influx case, and there was a significant overall decrease in the number of cancer cells in the domain. As a result of these influxes, most cells and cytokines were focused near the boundary of the tumor. Interestingly, despite the significant presence of the immune cells near the boundary, cancer cells have a higher concentration there. We hypothesize that the interactions resulting from the immune cells’ influxes create an outward divergence for the velocity field. This drives many of the cancer cells to the boundary of the domain. Since mitotic regions are usually close to the tumor boundary [106,111], this might explain why tumor cells can still escape after immunotherapy.

We calculated the sensitivity of total cancer cells to all the model parameters. The most sensitive reaction parameters are in agreement with the ODE study. Importantly, we observe a significant sensitivity to the diffusion coefficient of cancer cells and adipocytes. The interaction between cancer cells and adipocytes promotes cancer proliferation, and increasing these values elevates their interaction, leading to more cancer cells.

The 10% perturbation of the top 20 most sensitive parameters shows a small cancer growth at the lower bound, primarily due to the diffusion rates of cancer cells and adipocytes. Therefore, controlling the motility of these cells can lead to better prognoses, and there are already studies targeting cancer cells’ motility [118,119]. The results also indicate that the influx rate and source of cytotoxic T cells have a high impact on the total cancer cells. In other words, increased influx of these cells leads to fewer cancer cells, which is consistent with clinical and experimental observations [120,121,122]. Another important observation is the positive sensitivity values for the influx rate and source of macrophages. This is because macrophages produce IL6 and IL10, promoting cancer proliferation and inhibiting cytotoxic T cell proliferation. This result is also in line with biological and biomedical findings on the tumor-promoting properties of macrophages [114,115,116].

The findings of this study have to be seen in light of some limitations. One of the main challenges of mathematical modeling of cancer is the lack of data for more reliable parameter estimation and validation. Although we used the reaction parameters from the earlier ODE study, we did not have access to time-course data for this model to obtain the PDE-specific parameters. Therefore, we had to refer to studies that had estimated these parameters based on certain assumptions. Moreover, the cell-cell adhesion force value for the mechanical problem was a crude estimate. Currently, there is no direct report of such values in the literature to the best of the authors’ knowledge. In addition, nutrients and metabolites can significantly affect the tumor shape, size and number of cells. In avascular tumor models, the type of nutrients and the competition for their consumption between normal cells and cancer cells will lead to different growth pattern tendencies: circular or papillary-like [125]. On the other hand, depletion of nutrients and metabolites in the tumor microenvironment can cause the immune cells to lose their functionality, which can lead to cancer cells’ growth and immune invasion behavior [126]. However, to reduce the complexity of the model, we did not consider metabolites such as oxygen and nutrients.

Nevertheless, the model unveils some spatial features of breast tumor growth and identifies the most sensitive parameters, including diffusion rates of cancer cells and adipocytes to control the tumor growth. With access to more initial spatial data, this model can make predictions about the effect of early immune cells’ infiltration patterns on cancer progression. Additionally, future research can build upon this model to overcome its limitations, such as by including more biological processes or integrating different treatment options.

## Figures and Tables

**Table 1:** **PDE and ODE variables.** This table shows the relationship between the variables from (1) and the system of ODEs in [75].

Variable in PDE	Variable in ODE	Name
$X_{1}$	$T_{N}$	Naive T cells
$X_{2}$	$T_{h}$	Helper T cells
$X_{3}$	$T_{C}$	Cytotoxic cells
$X_{4}$	$T_{r}$	Regulatory T cells
$X_{5}$	$D_{N}$	Naive dendritic cells
$X_{6}$	*D*	Activated dendritic cells
$X_{7}$	$M_{N}$	Naive macrophages
$X_{8}$	*M*	Activated macrophages
$X_{9}$	*C*	Cancer cells
$X_{10}$	*N*	Necrotic cells
$X_{11}$	*A*	Cancer associated Adipocytes
$X_{12}$	*H*	HMGB1
$X_{13}$	$IL_{12}$	IL-12
$X_{14}$	$IL_{10}$	IL-10
$X_{15}$	$IL_{6}$	IL-6

**Table 2:** **Biomarker combinations.** (+) means high expression and (−) means lack of expression of a protein at a certain location.

Cell Type	Biomarker Combination
Helper T cells ($T_{h}$)	Epcam(−) CD45(+) CD3(+) CD4(+) CD8(−)
Cytotoxic T cells ($T_{C}$)	Epcam(−) CD45(+) CD3(+) CD4(−) CD8(+)
Naive dendritic cells ($D_{N}$)	Epcam(−) CD45(+) F4/80(−) CD11C(+)
Dendritic cells (*D*)	Epcam(−) CD45(+) F4/80(−) CD11C(+) MHC-II(+)
Activated macrophages (*M*)	Epcam(−) CD45(+) F4/80(+) CD11C(−) CSF1R(+) or Epcam(−) CD45(+) F4/80(+) CD11C(−) CSF1R(−) MHC-II(+)
Cancer cells (*C*)	Epcam(+) CD45(−)
Necrotic cells (*N*)	CC3(+)

## Data Availability

All the codes and data is available at https://github.com/ShahriyariLab/A-Bio-Mechanical-PDE-model-of-breast-tumor-progression-in-MMTV-PyMT-mice, accessed on 1 May 2022.

## Appendix A

In [75], we proposed an ODE model based on the interaction network in Figure 1. This model and its parameters were extracted from three time-course data acquired from three PyMT mouse models [127]. Figure A1 shows the results of the ODE model for all three mice.

## Appendix B

For the sample collection, tumor size was between 3–5 mm (early) and 12–15 mm in the longest dimension (late) at the time of excision. Tumors were immediately fixed in 10% formalin for 48 h, then perfused with paraffin. Specimen were sectioned using a standard microtome to collect 4–5 μm tissue sections. Dry FFPE tissues were baked in a 65 ∘C oven for 30 min, followed by deparaffinization with xylene and rehydration in serially graded alcohol to water. Sections were then stained by multiplex immunohistochemistry.

The slides were subjected to staining with F4/80 and CSF1R antibodies (no antigen retrieval) and hematoxylin staining (S3301, Dako, Santa Clara, CA, USA). Then, we subjected them to the first heat-mediated antigen retrieval in 1× pH 5.5–6 citrate buffer for 90 s in a low-power microwave and 16 min in a steamer. This step was followed by iterative cycles of (i) staining, (ii) whole slide scanning, and (iii) and antibody stripping. Slides were incubated with primary antibodies (as defined in Tatarova et al., manuscript under review) for 1 h at RT or overnight at 4 degrees Celsius in a humid chamber. Signal was visualized with either anti-rabbit or anti-rat Histofine Simple Stain MAX PO horseradish peroxidase-conjugated polymer followed by peroxidase detection with 3-amino-9-ethylcarbazole (AEC). While agitating, all washing steps were performed three times for 5–10 min in 1× PBS. Images were acquired using the Aperio ImageScope AT (Leica Biosystems) at 20× magnification. Within one cycle, AEC and HRP inactivation removal was accomplished by incubating the slides in 0.6% fresh H_2_O_2_ in methanol for 15 min within one cycle; AEC removal and stripping of antibodies was accomplished by ethanol gradient incubation and heat-mediated antigen retrieval between cycles. After washing, samples were subjected to the subsequent staining round.

The iteratively digitized images were co-registered using Matlab (The MathWorks, Inc., Natick, MA, USA, version 2019b). The imperfectly registered images were additionally processed using Fiji’s Linear Stack Alignment with SIFT plugin. Hematoxylin-stained images served for single-cell nuclear segmentation to generate a binary mask using the watershed in Fiji. Target protein signal was extracted using the NIH plugin RGB_to_CMYK to separate the AEC signal into the yellow channel for improved sensitivity of IHC evaluation. Grayscale images of all proteins and the binary mask were imported to CellProfiler (version 3.1.8, Broad Institute, Cambridge, MA, USA) to quantify single-cell signal mean intensity per mask. The mean signal intensity per segmented cell was imported to FCS Express 6 and 7 Image Cytometry Software (DeNovo Software). Single-cell data from a single random intratumoral region (per stage) were extracted from FCS Express in the data grid for downstream modeling. The quality of the single-cell data was ensured by excluding folded or lost tissue.

## Appendix C

Figure A2 shows the initial condition preparation steps.

## Appendix D

### Appendix D.1. Weak Formulation and Discretization

Suppose ξ and *q* are proper arbitrary vector and scalar test functions, respectively; then multiplying both sides of Equations (9) and (11) by these functions and using (12) along with integration by parts gives

(A1) $$\begin{array}{c} F\left(v,p,\xi ,q\right)=\int_{\Omega (t)}Q:\nabla \xi \;dx+\int_{\Gamma (t)}\gamma \kappa n\cdot \xi \;ds+\int_{\Omega (t)}\left(\nabla \cdot v-\frac{\sum_{i\in I}f_{i}}{\sum_{i\in I}\left[X_{i}\right]}\right)q\;dx=0. \end{array}$$

To exclude rigid body movements, we consider the following constraints with Lagrange multipliers $\lambda_{i}$ and test functions $\omega_{i}$ for i=1,2,3 corresponding to the bases $z_{i}$ of the space of rigid body movements:

(A2) $$\begin{array}{c} G\left(v,\lambda_{i},\xi ,\omega_{i}\right)=\int_{\Omega (t)}\left[\sum_{i=1}^{3}\lambda_{i}\left(\xi \cdot z_{i}\right)\right]\;dx+\int_{\Omega (t)}\left[\sum_{i=1}^{3}\omega_{i}\left(v\cdot z_{i}\right)\right]\;dx=0, \end{array}$$

where $z_{i}\in \left\{\left(1,0\right),\left(0,1\right),\left(-y,x\right)\right\}$. Therefore, we find v, *p* and $\lambda_{i}$ that satisfy

(A3) $$\begin{array}{c} F\left(v,p,\xi ,q\right)-G\left(v,\lambda_{i},\xi ,\omega_{i}\right)=0,\;\forall \;\xi ,q,\omega_{i}. \end{array}$$

We then make sure that v satisfies (13) when we move the mesh. Before discussing the weak form of the system of equations in (1), we rewrite them in the Eulerian from:

(A4) $$\begin{array}{c} \frac{D[X_{i}]}{Dt}+b_{i}\;\left[X_{i}\right]\nabla \cdot v=D_{i}\Delta \left[X_{i}\right]+f_{i}. \end{array}$$

The differential operator $\frac{D}{Dt}=\frac{d}{dt}+v\cdot \nabla$ is the material derivative. This setup will make the numerical simulations much easier, since we can always solve (A4) in the current domain configuration. For appropriate test functions $\zeta_{i}$ the weak form is given by

(A5) $$\begin{array}{ccc} H(\left[X_{i}\right],\zeta_{i},\frac{D[X_{i}]}{Dt}) & = & \sum_{i=1}^{15}\left[\int_{\Omega (t+1)}\left(\frac{D[X_{i}]}{Dt}+b_{i}\;\left[X_{i}\right]\nabla \cdot v-f_{i}\right)\;\zeta_{i}\;dx\right]\\ & & +\sum_{i=1}^{15}\left[\int_{\Omega (t+1)}D_{i}\left(\nabla \left[X_{i}\right]\cdot \nabla \zeta_{i}\right)\;dx\right]\\ & & +\sum_{i=1}^{15}\left[\int_{\Gamma (t+1)}\alpha_{i}\left(\left[X_{i}\right]-\left[{X_{i}}^{*}\right]\right)\;\zeta_{i}\;ds\right]=0. \end{array}$$

For discretization of the mechanical problem, we use the mixed finite element space $W_{h}^{t}$ defined by

(A6) $$\begin{array}{c} W_{h}^{t}=\left\{\left(v_{h}^{t},p_{h}^{t},\Lambda \right)|u_{h}^{t}\in V_{h}^{t},p_{h}^{t}\in S_{h}^{t},\Lambda =\left(\lambda_{1},\lambda_{2},\lambda_{3},\lambda_{4}\right)\in R^{4}\right\}. \end{array}$$

We take the space $V_{h}^{t}$ to be a piece-wise quartic vector function space and $S_{h}^{t}$ a piece-wise linear function space defined on $\Omega_{h}^{t}$ (the triangulated discrete mesh representing Ω(t)). If we denote the number of triangles in $\Omega_{h}^{t}$ by *N* and the basis functions for $V_{h}^{t}$ and $S_{h}^{t}$ by ${\left\{w_{i}^{t}\right\}}_{i=1}^{N}$ and ${\left\{s_{i}^{t}\right\}}_{i=1}^{N}$, respectively, then

(A7) $$\begin{array}{c} v_{h}^{t}=\sum_{i=1}^{N}c_{i}^{t}w_{i}^{t}\;,\;p_{h}^{t}=\sum_{i=1}^{N}k_{i}^{t}s_{i}^{t}. \end{array}$$

We do not invent new notations for test functions and assume they are always chosen appropriately according to each space. Now, we solve the discrete version of (A3) on $\Omega_{h}^{t}$ and its boundary $\Gamma_{h}^{t}$

(A8) $$\begin{array}{c} F\left(v_{h}^{t},p_{h}^{t},\xi ,q\right)-G\left(v_{h}^{t},\lambda_{i},\xi ,\omega_{i}\right)=0,\;\forall \;\xi ,q,\omega_{i} \end{array}$$

which gives us the solution ${\bar{v}}_{h}^{t}$. Assuming that our time step sizes are dt, then

(A9) $$\begin{array}{c} {\bar{v}}_{h}^{t}\approx \frac{{\bar{u}}_{h}^{t}-{\bar{u}}_{h}^{t-1}}{dt}. \end{array}$$

Moving nodes of $\Omega_{h}^{0}$ by the displacement ${\bar{u}}_{h}^{t-1}$ produces $\Omega_{h}^{t}$, and moving $\Omega_{h}^{0}$ via ${\bar{u}}_{h}^{t}$ gives $\Omega_{h}^{t+1}$. Therefore, for going from $\Omega_{h}^{t}$ to $\Omega_{h}^{t+1}$ we can use the flow map $x\left(x_{h}^{0},t+1\right)=x\left(x_{h}^{0},t\right)+{\bar{U}}_{h}^{t}$ where ${\bar{U}}_{h}^{t}={\bar{u}}_{h}^{t}-{\bar{u}}_{h}^{t-1}$. We use a forward Euler to discretize the material derivative.

(A10) $$\begin{array}{c} \frac{D[X_{i}]}{Dt}\approx D_{h,dt}\left[X_{i}\right]:=\frac{\left[X_{i}\right]\left(x\left(x_{h}^{0},t+1\right),t+1\right)-\left[X_{i}\right]\left(x\left(x_{h}^{0},t\right),t\right)}{dt}. \end{array}$$

Here, we should point out that there is no need for interpolation in (A10) since the mesh points move with the flow map.

Equation (A4) corresponding to cell dynamics has very small diffusion coefficients (see (4)). Therefore, they can become advection or reaction-dominated, resulting in numerical instabilities. We enriched the usual piece-wise linear function spaces with bubble elements to remedy this. This method allows us to increase the stability without increasing the size of the problem significantly [128,129,130]. Therefore, on the moved mesh $\Omega_{h}^{t+1}$ we define bubble enriched piece-wise linear function spaces $Q_{h,B}^{t+1}=Q_{h}^{t+1}⨁Q_{B}^{t+1}$, and use the following mixed space

(A11) $$\begin{array}{c} M_{h}^{t+1}=\left\{\left({\left[X_{1}\right]}_{h}^{t+1},\cdots ,{\left[X_{15}\right]}_{h}^{t+1}\right)\;|\;{\left[X_{1}\right]}_{h}^{t+1}\in Q_{h,B}^{t+1}\right\}, \end{array}$$

and we solve

$$H({\left[X_{i}\right]}_{h}^{t+1},\zeta_{i},D_{h,dt}{\left[X_{i}\right]}_{h}^{t+1})=0$$

for cells and molecules concentrations in the current domain.

### Appendix D.2. Simulation

The simulations in this study were carried out via FEniCS [131], and all the meshes were generated via GMSH (http://gmsh.info/, accessed on 1 March 2022). Both of these software packages are very powerful. With FEniCS, we can easily work with mixed, enriched, and higher-order spaces without the need to write numerous lines of code. GMSH can easily handle structured and non-structured domains with several sub-domains and boundary surfaces. Additionally, it can be called from within FEniCS for more flexible re-meshing and has many other useful features.

## Appendix E

Table A1 contains a full sensitivity report of the PDE parameters in addition to the reaction parameters used in the ODE study [75].

**Table A1:** **Full sensitivity report.** This table contains the sensitivity value of $\int_{\Omega }Cdx$ to all the parameters used in the model. The rows are ordered in a decreasing fashion based on the absolute value of their sensitivity values.

Order	Notation	Sensitivity	Definition	Order	Notation	Sensitivity	Definition
1	$D_{C}$	2242.835	Diffusion coefficient of *C*	44	$\delta_{T_{h}}$	−$2.87\times 10^{-5}$	Death rate of $T_{h}$
2	$D_{A}$	56.46447	Diffusion coefficient of *A*	45	$\lambda_{MT_{h}}$	$2.10\times 10^{-5}$	Activation rate of *M* by $T_{h}$
3	$\delta_{C}$	−3.16066	Death rate of *C*	46	$A_{D_{N}}$	−$1.48\times 10^{-5}$	Independent production rate of $D_{N}$
4	$\lambda_{C}$	2.989047	Proliferation rate of *C*	47	$\delta_{T_{N}}$	$1.41\times 10^{-5}$	Death rate of $T_{N}$
5	$\alpha_{T_{C}}$	−0.31011	Influx rate of $T_{C}$	48	$\lambda_{T_{h}H}$	$1.03\times 10^{-5}$	Activation rate of $T_{h}$ by *H*
6	$D_{T_{C}}$	−0.2628	Diffusion coefficient of $T_{C}$	49	$\lambda_{DC}$	−$6.42\times 10^{-6}$	Activation rate of *D* by *C*
7	$\lambda_{CIL_{6}}$	0.138431	Proliferation rate of *C* by $IL_{6}$	50	$\lambda_{T_{h}IL_{12}}$	$6.27\times 10^{-6}$	Activation rate of $T_{h}$ by $IL_{12}$
8	$\lambda_{CA}$	0.107539	Proliferation rate of *C* by *A*	51	$A_{M_{N}}$	$3.50\times 10^{-6}$	Independent production rate of $M_{N}$
9	$\delta_{CT_{C}}$	−0.06665	Inhibition rate of *C* by $T_{C}$	52	$\lambda_{T_{C}D}$	−$3.46\times 10^{-6}$	Activation rate of $T_{C}$ by *D*
10	$\delta_{A}$	−0.03611	Death rate of *A*	53	$\delta_{D_{N}}$	$3.26\times 10^{-6}$	Death rate of $D_{N}$
11	$\lambda_{A}$	0.03531	Proliferation rate of *A*	54	$\lambda_{IL_{10}D}$	$3.14\times 10^{-6}$	Production rate of $IL_{10}$ by *D*
12	$\alpha_{M}$	0.025664	Influx rate of *M*	55	$\delta_{T_{h}IL_{10}}$	−$1.71\times 10^{-6}$	Inhibition rate of $T_{h}$ by $IL_{10}$
13	$\delta_{IL_{6}}$	−0.01152	Decay rate of $IL_{6}$	56	$\delta_{T_{h}T_{r}}$	−$1.46\times 10^{-6}$	Inhibition rate of $T_{h}$ by $T_{r}$
14	$\lambda_{IL_{6}A}$	0.009239	Production rate of $IL_{6}$ by *A*	57	$\lambda_{DH}$	−$1.37\times 10^{-6}$	Activation rate of *D* by *H*
15	$\lambda_{IL_{6}M}$	0.007813	Production rate of $IL_{6}$ by *M*	58	$A_{0}$	$9.75\times 10^{-7}$	Carrying capacity of *A*
16	$\alpha_{T_{h}}$	0.002154	Influx rate of $T_{h}$	59	$\delta_{M_{N}}$	−$3.58\times 10^{-7}$	Death rate of $M_{N}$
17	$\delta_{T_{C}}$	0.001508	Death rate of $T_{C}$	60	$\lambda_{IL_{12}M}$	−$3.55\times 10^{-7}$	Production rate of $IL_{12}$ by *M*
18	$\alpha_{T_{r}}$	0.001441	Influx rate of $T_{r}$	61	$\delta_{IL_{12}}$	$2.87\times 10^{-7}$	Decay rate of $IL_{12}$
19	$D_{D_{N}}$	−0.00094	Diffusion coefficient of $D_{N}$	62	$\lambda_{IL_{12}T_{h}}$	−$2.31\times 10^{-7}$	Production rate of $IL_{12}$ by $T_{h}$
20	$D_{N}$	−0.00094	Diffusion coefficient of *N*	63	$\lambda_{IL_{12}T_{C}}$	−$2.18\times 10^{-7}$	Production rate of $IL_{12}$ by $T_{C}$
21	$C_{0}$	0.000541	Carrying capacity of *C*	64	$D_{IL_{12}}$	9.50×106−8	Diffusion coefficient of $IL_{12}$
22	$D_{T_{r}}$	−0.00044	Diffusion coefficient of $T_{r}$	65	$\lambda_{T_{h}D}$	$7.78\times 10^{-8}$	Activation rate of $T_{h}$ by *D*
23	$\delta_{IL_{10}}$	−0.00039	Decay rate of $IL_{10}$	66	$\lambda_{T_{r}D}$	$6.93\times 10^{-8}$	Activation rate of $T_{r}$ by *D*
24	$\lambda_{IL_{10}C}$	0.000386	Production rate of $IL_{10}$ by *C*	67	$\delta_{D}$	−$3.70\times 10^{-8}$	Death rate of *D*
25	$\lambda_{T_{C}IL_{12}}$	−0.00035	Activation rate of $T_{C}$ by $IL_{12}$	68	$\delta_{H}$	−$1.49\times 10^{-8}$	Decay rate of *H*
26	$D_{M}$	−0.00034	Diffusion coefficient of *M*	69	$\lambda_{HM}$	$8.05\times 10^{-9}$	Production rate of *H* by *M*
27	$\lambda_{IL_{10}T_{r}}$	0.000319	Production rate of $IL_{10}$ by $T_{r}$	70	$\lambda_{HT_{C}}$	$5.35\times 10^{-9}$	Production rate of *H* by $T_{C}$
28	$\delta_{M}$	−0.00029	Death rate of *M*	71	$\lambda_{HC}$	$5.00\times 10^{-9}$	Production rate of *H* by *C*
29	$\lambda_{IL_{10}M}$	0.00024	Production rate of $IL_{10}$ by *M*	72	$\delta_{DC}$	$3.46\times 10^{-9}$	Activation rate of *D* by *C*
30	$\lambda_{IL_{10}T_{h}}$	0.00018	Production rate of $IL_{10}$ by $T_{h}$	73	$\lambda_{IL_{12}D}$	−$2.50\times 10^{-9}$	Production rate of $IL_{12}$ by *D*
31	$\lambda_{IL_{10}T_{C}}$	0.000153	Production rate of *IL*_10_ by $T_{C}$	74	$\lambda_{HN}$	$4.23\times 10^{-10}$	Production rate of *H* by *N*
32	$D_{T_{h}}$	−0.00015	Diffusion coefficient of $T_{h}$	75	$\lambda_{HD}$	$1.96\times 10^{-10}$	Production rate of *H* by *D*
33	$D_{IL_{6}}$	0.000126	Diffusion coefficient of $IL_{6}$	76	$\delta_{N}$	$1.14\times 10^{-11}$	Death rate of *N*
34	$A_{T_{N}}$	−$9.73\times 10^{-5}$	Independent production rate of $T_{N}$	77	$D_{H}$	$1.90\times 10^{-13}$	Diffusion coefficient of *H*
35	$\alpha_{D_{N}}$	$9.34\times 10^{-5}$	Influx rate of $D_{N}$	78	$\alpha_{NC}$	$8.36\times 10^{-15}$	*C* to *N* conversion fraction
36	$\delta_{T_{C}T_{r}}$	$8.92\times 10^{-5}$	Inhibition rate of $T_{C}$ by $T_{r}$	79	$T_{C}^{*}$	−$3.56\times 10^{-16}$	$T_{C}$ influx source
37	$D_{D}$	$8.69\times 10^{-5}$	Diffusion coefficient of *D*	80	$M^{*}$	$3.44\times 10^{-17}$	*M* influx source
38	$\delta_{T_{C}IL_{10}}$	$7.88\times 10^{-5}$	Inhibition rate of $T_{C}$ by $IL_{10}$	81	$T_{h}^{*}$	$2.87\times 10^{-18}$	$T_{h}$ influx source
39	$\lambda_{IL_{6}D}$	$7.41\times 10^{-5}$	Production rate of $IL_{6}$ by *D*	82	$T_{r}^{*}$	$2.47\times 10^{-18}$	$T_{r}$ influx source
40	$D_{IL_{10}}$	−$5.72\times 10^{-5}$	Diffusion coefficient of $IL_{10}$	83	$D_{N}^{*}$	−$8.55\times 10^{-20}$	$D_{N}$ influx source
41	$\lambda_{MIL_{10}}$	$5.35\times 10^{-5}$	Activation rate of *M* by $IL_{10}$				
42	$\delta_{T_{r}}$	−$4.66\times 10^{-5}$	Death rate of $T_{r}$				
43	$\lambda_{MIL_{12}}$	$3.41\times 10^{-5}$	Activation rate of *M* by $IL_{12}$				
